# Fibroblast viability and phenotypic changes within glycated stiffened three-dimensional collagen matrices

**DOI:** 10.1186/s12931-015-0237-z

**Published:** 2015-07-01

**Authors:** Vanesa Vicens-Zygmunt, Susanna Estany, Adai Colom, Ana Montes-Worboys, Carlos Machahua, Andrea Juliana Sanabria, Roger Llatjos, Ignacio Escobar, Frederic Manresa, Jordi Dorca, Daniel Navajas, Jordi Alcaraz, Maria Molina-Molina

**Affiliations:** Department of Pneumology, Unit of Interstitial Lung Diseases, University Hospital of Bellvitge, Barcelona, Spain; Pneumology Research Group, IDIBELL, University of Barcelona, Barcelona, Spain; Unit of Biophysics and Bioengineering, University of Barcelona, Barcelona, Spain; Department of Preventive Medicine, University Hospital of Bellvitge, Barcelona, Spain; Department of Pathology, University Hospital of Bellvitge, Barcelona, Spain; Department of Thoracic Surgery, University Hospital of Bellvitge, Barcelona, Spain; Research Network in Respiratory Diseases (Centro de Investigación Biomédica en Red (CIBER) de Enfermedades Respiratorias), ISCIII, Barcelona, Spain; Department of Biochemistry, University of Geneva, Science II, Geneva, Switzerland

**Keywords:** Three-dimensional matrices, Collagen, Fibroblasts, Alpha-smooth muscle actin, Non-enzymatic glycation, Advanced glycation end products (AGEs), Viability, Stiffness, Contractility, Lung fibrosis

## Abstract

**Background:**

There is growing interest in the development of cell culture assays that enable the rigidity of the extracellular matrix to be increased. A promising approach is based on three-dimensional collagen type I matrices that are stiffened by cross-linking through non-enzymatic glycation with reducing sugars.

**Methods:**

The present study evaluated the biomechanical changes in the non-enzymatically glycated type I collagen matrices, including collagen organization, the advanced glycation end products formation and stiffness achievement. Gels were glycated with ribose at different concentrations (0, 5, 15, 30 and 240 mM). The viability and the phenotypic changes of primary human lung fibroblasts cultured within the non-enzymatically glycated gels were also evaluated along three consecutive weeks. Statistical tests used for data analyze were Mann–Whitney U, Kruskal Wallis, Student’s *t*-test, two-way ANOVA, multivariate ANOVA, linear regression test and mixed linear model.

**Results:**

Our findings indicated that the process of collagen glycation increases the stiffness of the matrices and generates advanced glycation end products in a ribose concentration-dependent manner. Furthermore, we identified optimal ribose concentrations and media conditions for cell viability and growth within the glycated matrices. The microenvironment of this collagen based three-dimensional culture induces α-smooth muscle actin and tenascin-C fibroblast protein expression. Finally, a progressive contractile phenotype cell differentiation was associated with the contraction of these gels.

**Conclusions:**

The use of non-enzymatic glycation with a low ribose concentration may provide a suitable model with a mechanic and oxidative modified environment with cells embedded in it, which allowed cell proliferation and induced fibroblast phenotypic changes. Such culture model could be appropriate for investigations of the behavior and phenotypic changes in cells that occur during lung fibrosis as well as for testing different antifibrotic therapies *in vitro*.

**Electronic supplementary material:**

The online version of this article (doi:10.1186/s12931-015-0237-z) contains supplementary material, which is available to authorized users.

## Background

Pulmonary extracellular matrix (ECM) is a complex mesh of proteins, proteoglycans and glycosaminoglycans, which contains the cells and participates in tissue’s homeostasis and repair. Active lung fibroblasts, myofibroblasts and other cell types, are normally surrounded by this fibrous three-dimensional (3D) extracellular matrix and are mainly responsible for the synthesis, secretion and degradation of the ECM components. Moreover, these cells are responsible for the correct turnover of ECM proteins, preserving the lung architecture and function [1]. However, most *in vitro* studies of fibroblasts behavior have been performed using conventional two-dimensional (2D) cultures, which lack the structural three-dimensionality provided by the ECM in the original tissue. This limitation is important because it can cause the loss or change of important tissue-specific cell functions due to the lack of essential ECM signals [2, 3]. A suitable alternative to overcome this limitation is 3D cultures, in which cells are embedded within ECM gels. Studies using 3D cultures have shown that the ECM influences cell behavior through mechanical interactions and biochemical signals [3–6]. Moreover, these studies have clearly shown that cell functions and phenotypes depend on the structure of the microenvironment, such as the ECM [2–4] and have revealed the profound impact of extracellular forces on the behavior of fibroblasts and other cell types [3, 7, 8].

On the other hand, fibrotic lung diseases, such as idiopathic pulmonary fibrosis (IPF), are characterized by an excessive ECM deposition and the expansion of the fibroblast/myofibroblast population leading to an increased parenchyma stiffness and retraction, with final lung destruction and patient death [9–12]. In this context, the increased matrix stiffening observed in the lung fibrotic process may be a critical fibrogenesis driving factor [13, 14]. Unfortunately, suitable 3D culture assays for the study of fibrotic lung diseases and other aging disorders, primarily mimicking matrix stiffness are scanty.

Therefore, there is growing interest in developing 3D culture assays that enable increasing the rigidity of the ECM mimicking the situation observed during fibrotic processes *in vivo*. In some 3D culture assays based on collagen gels, the rigidity of the ECM is modified by changing the ECM concentration [15]. Alternatively, ECM stiffness can be changed by increasing the cross-linking through non-enzymatic glycation with reducing sugars, which cause the accumulation of the final products of glycation, i.e., the advanced glycation end products (AGEs) [3, 5, 16–24]. Interestingly, an excess of AGEs in aged human tissues has been observed to stiffen tissues in different degenerative diseases [25] and may accelerate protein oxidation, altering their structure and threatening their function [23, 24]. Therefore, various physical characteristics of the fibrotic lung tissue, such as reduced elastic recoil [2], decreased matrix degradation [21, 22, 26, 27], oxidative ECM protein damage [28], may be related to the accumulation of AGEs, contributing to the acceleration of the fibrotic process. However, the effects of non-enzymatic glycation on collagen gels and the resulting behavior of lung fibroblasts remain largely unknown.

Thus, the aims of this study were: 1) to develop an experimental culture model system that recreate the biomechanical and three-dimensional conditions of fibrotic lungs, and 2) to examine the cellular viability and biomechanical changes in the ECM, including AGE formation and stiffening, in these non-enzymatically glycated collagen gels. This model may facilitate the experimental study of the complex cell-extracellular pro-fibrotic interactions, such as those occurring in fibrotic lung diseases, as IPF.

## Methods

### Development of non-cellular post-glycated three-dimensional matrix

The 3D collagen matrices were produced using native type I collagen from bovine dermis (AteloCell, KOKEN, Japan), following a previously standardized protocol [16] with some modifications. Briefly, type I collagen (4 mg/ml), 1x Dulbecco’s modified Eagle medium (DMEM 10x, Sigma-Aldrich, Germany) or 1x phosphate-buffered saline (PBS 10x, Lonza, Switzerland) were mixed on ice, and then the pH was carefully adjusted to 7.4 ± 0.2 using sterile sodium hydroxide (Sigma-Aldrich). Subsequently, the gels were polymerized at 37 °C for 30 minutes.

The glycated gels were prepared using a non-enzymatic post-glycation method to modify the biomechanics properties of collagen. Ribose (D-Ribose, Sigma-Aldrich) was used to glycate the matrices according to the literature [19–22]. After gels polymerization, different ribose concentrations (0, 30 and 240 mM) were added in the media (day 0), which were not changed until the fifth day to allow cross-linking between the collagen and the reducing sugar to occur [19–21, 27]. Then, medium without ribose was used, which was changed every two days for twenty-one consecutive days.

### Physical characteristics of the non-cellular post-glycated three-dimensional matrix

#### Confocal reflectance collagen imaging

Collagen gels under all of the ribose conditions were prepared in 35 mm glass-bottom culture dishes (MatTek, USA). The collagen structure was examined using confocal reflection microscopy (CRM) as reported elsewhere [29]. In brief, reflection fluorescence of collagen fibers were visualized by exciting the collagen gels using a 568 nm laser and collecting the reflected signal of the same wavelength. Images were acquired on the 7^th^, 14^th^ and 21^st^ days post-polymerization with an inverted confocal laser-scanning microscope (D-Eclipse C1, Nikon, Tokyo, Japan) coupled to an EM-CCD digital camera (C9100, Hamamatsu, Japan) equipped with an ELWD 60×/0.70 objective (Nikon, Tokyo, Japan) using Metamorph software (Molecular Devices, USA). The same image acquisition parameters were used for all of the gels at each time point. The images were analyzed using Image J program [30].

#### Advanced glycation end products (AGEs)

Different experimental conditions were used to produce the post-glycated DMEM and PBS three-dimensional collagen matrices as follows: ribose at 0, 5, 15, 30 or 240 mM and Fetal Bovine Serum (FBS) at 0, 1 or 10 % was added to the media on the day 0. The level of fluorescent advanced glycation end products (AGEs) generated in collagen matrices was determined by an indirect measure in dark 96-well sterile plates (Nunc, Denmark) using a FLUOstar OPTIMA plate reader (BMG Labtech, Germany) at ex/em: 360/440 nm wavelength [20, 21, 27, 28, 31, 32], on the 1^st^, 7^th^, 14^th^ and 21^st^ days post-polymerization. The level of matrices and media autofluorescence was individually analyzed.

#### Gel mechanical measurements by atomic force microscopy (AFM)

Post-glycated 3D collagen matrices were elaborated as explained above using the same conditions of collagen fibers configuration (confocal reflectance imaging). The ribose-induced mechanical alterations in the collagen gels were examined on the 7^th^, 14^th^ and 21^st^ days by assessing their Young’s elastic modulus, *E*, which is indicative of gel’s resistance to deformation. *E* was measured using a stand-alone AFM (Bioscope, Veeco, USA) coupled to an inverted optical microscope as previously described [33]. Briefly, the AFM measurements were taken using low spring constant cantilevers with pyramidal tips (nominal *k* = 0.01 N/m) (Microlever, Veeco), which were calibrated using the thermal noise method [34]. For each gel location, three force vs. piezo displacement curves (*F*-*z* curves) were acquired using a moderate loading force (~1 nN). For each gel location, *E* and the extent of gel indentation (*d*) were computed as the averages of the values obtained by least-squares fitting of a contact elastic model to each set of three *F-z* curves, as reported elsewhere [35]. The same protocol was applied on at least 9 random gel locations. For each treatment, the *E* data were normalized using the corresponding value obtained from 0 mM ribose gel at the 7^th^ day.

### Effects of post-glycated matrices on primary human normal lung fibroblasts

#### Ethics Statement

This study involved the analysis of human clinical samples. Normal human lung tissues were obtained from lung biopsies from patients with pneumothorax and no history of tobacco use, diabetes mellitus or any other pathology and that were histologically normal and without infection. Informed written consent was obtained from all patients. The investigation project and protocol were approved by the Ethics Committee of our center (ref. PR202/08, CEIC, Institute of Investigation of Bellvitge (IDIBELL), Barcelona, Spain).

#### Cell culture

The primary human normal lung fibroblasts were isolated from lung biopsies, following the standard methods described in the literature [36]. Briefly, 1 mm^2^ fragments of tissue were incubated under sterile conditions in DMEM supplemented with 10 % FBS (Gibco, UK), 100 IU/mL penicillin (Gibco), 100 μg/mL streptomycin (Gibco) and 2 μg/mL amphotericin B (Sigma-Aldrich). The fibroblasts cultures were maintained in a 5 % CO_2_ humidified atmosphere at 37 °C. After two-three weeks of culture cells were attached to the plate. Unattached cells were eliminated by changing the media. When reaching the confluence, the morphology of cells were fibroblasts type, and resulted positive for vimentin and negative for alpha-sma markers, indicating the mesenchymal origin (Additional file 1). Fibroblasts were then trypsinized (0.05 % trypsin-EDTA, Gibco) and cryopreserved using standard methods and used at passages (3–5) for culturing within the post-glycated matrices.

#### Fibroblasts culture within the post-glycated three-dimensional collagen type I matrices

Type I collagen matrices were prepared as explained before. The primary normal fibroblasts were added and mixed with the collagen before polymerization of the gel. Cells were cultured in 3D post-glycated DMEM collagen matrices in sterile 96-well plates (TPP, Switzerland) at an initial concentration of 15x10^3^ cells/gel. After polymerization of the matrices, media with different concentration of ribose and FBS was added (5, 15, 30 and 240 mM of ribose and 0, 1 and 10 % of FBS). Cell viability and phenotype changes were evaluated on the 7^th^, 14^th^ and 21^st^ days of culture. Analyze of cell viability was adjusted to day 1 and the results were shown since the 7^th^ day to allow cell stabilization and adaptation into the glycated 3D gels. The results were evaluated from the 7^th^ day at different time-points and conditions because until the 5^th^ day the media was not changed to permit collagen cross-links.

#### Fibroblast viability

The viability of the fibroblasts growing within the post-glycated 3D collagen type I matrices was assessed using a metabolic test (AlamarBlue assay, Invitrogen, UK) and a staining method that distinguishes viable and non-viable cells (LIVE/DEAD viability/cytotoxicity kit, Invitrogen) on the 7^th^, 14^th^ and 21^st^ days, following the manufacturer’s recommendations. The AlamarBlue assay, which is normally used in 2D cultures, required preliminary tests for use in the 3D collagen matrices. After testing AlamarBlue at several concentrations and incubation times for twenty-one days, incubation time of 8 h and concentration of 1/10 were used in all viability experiments. Then, the assay was conducted according to the manufacturer’s recommendations. The cell fluorescence was measured using a microplate reader (FLUOstar OPTIMA, BMG Labtech) at ex/em: 550/590 nm wavelengths. The LIVE/DEAD viability/cytotoxicity reagents stain viable cells in green (Calcein acetoxymethyl ester; Ca AM (ex 495/em 515 nm wavelengths)) and non-viable cells in red (ethidium homodimer-1; Eth-D1 (ex 495/em 635 nm wavelength). Images of stained sections were acquired using a Leica TCS-SL filter-free spectral confocal laser scanning microscope (Leica Microsystems, Mannheim, Germany) equipped with a 488 nm argon laser using a 10x dry objective (0.3 numerical aperture). Three-dimensional image assembly was performed using the fiji-win32 program (Image J). The cells were typified as live or dead using manual counting by two observers. An additional movie shows the cellular distribution in 3D-matrices after stained with Calcein and Ethidium stains (see Additional file 2). An additional image of fibroblasts morphology, when they are cultured into the DMEM and PBS matrices at different FBS concentrations (0, 1 and 10 %), is available as Additional file 3.

#### α-smooth muscle actin gene expression

RNA extraction was performed using the guanidiniumthiocyanate-phenol-chloroform method (TRIzol, Invitrogen), according to the manufacturer’s recommendation. First, the matrices were placed in TRIzol Reagent and disrupted using a tissue homogenizer. To eliminate the genomic DNA, the RNA was treated with DNAse I (Invitrogen). One microgram of total RNA was reverse transcribed to cDNA using a High Capacity cDNA Reverse Transcription kit (Applied Biosystems, UK). α-smooth muscle actin (α-SMA) gene expression was evaluated using real-time quantitative reverse transcription-polymerase chain reaction (qPCR) with the TaqMan Gene Expression master mix (7900 HT, Applied Biosystems). The results were analyzed by the ∆∆Ct method, normalized with two endogenous housekeeping genes (Eukaryotic 18S rRNA, 18S and DNA-directed RNA polymerase II, RPII).

#### α-smooth muscle actin Western Blot

Protein extraction from the matrices was performed according to the Heidebrecht protocol with few modifications [37]. The matrices were rinsed with iced-cold PBS, and the total cellular protein extracts were prepared using a radioimmunoprecipitation assay buffer (RIPA, Sigma-Aldrich) containing phosphatase and protease inhibitor cocktail (Sigma-Aldrich); the samples were sonicated and then frozen at −80 ° C until protein quantification was performed. Prior to performing the protein quantification, a 2-D clean-up (GE Healthcare Life Science, Germany) was conducted. Protein quantification was performed using a BCA Protein Assay Reagent (Thermo scientific, Germany) following the manufacturer’s instructions. Thirty micrograms of protein extract was loaded to SDS-polyacrylamide gel electrophoresis (SDS-PAGE, Mini-PROTEAN® TGX™ gels, Bio-Rad Laboratories, USA). The proteins were transferred to Trans-Blot® Turbo™ Mini Nitrocellulose Transfer Packs (Bio-Rad) using a Trans-Blot® Turbo™ Transfer Starter System (Bio-Rad). The membranes were blocked using 5 % BSA (bovine serum albumin, Sigma-Aldrich) in TBST (Tris-buffered saline with 0.1 % Tween 20) for 1 h; incubated with the primary antibodies: α-SMA (1:500, Sigma-Aldrich) and α-tubulin (1:1000, Sigma-Aldrich) for 1 h and finally incubated with a secondary anti-mouse IgG antibody conjugated with horseradish peroxidase (1:1000, HRP, Dako, Denmark) for 1 h at room temperature. After each antibody incubation step, the membranes were washed three times using TBST for 10 minutes each. The bands were visualized with enhanced chemiluminescence using Clarity™ Western ECL Substrate (Bio-Rad), and images were captured using a LAS-3000 Imaging System (Fujifilm Holdings Corporation, Japan). As a band of 66 KDa was always present in cellular and acellular matrices, an incubation with the three primary antibodies together for 1 h (bovine albumin (1:8000, Abcam), α-SMA (1:500, Sigma-Aldrich) and α-tubulin (1:1000, Sigma-Aldrich)) was performed confirming that the band observed at 66 KDa was albumin, probably due to the difficulties in rinsing all the media with FBS contained in the matrices prior to protein extraction (data not shown).

Western blot densitometry was performed using MultiGauge image software (Fujifilm) by normalizing the values for α-SMA expression to the respective value of α-tubulin for each sample.

#### Tenascin-C expression

To assess the expression of the pro-fibrotic protein Tenascin-C (TNC) produced by fibroblasts, an ELISA using a Human Tenascin-C Large Assay Kit (FN III-C, IBL International, Germany) was performed following the manufacturer’s instructions.

TNC was evaluated from the supernatants of the DMEM matrices when gel contraction was observed (i.e. when 10 % of FBS was used). The precoated plate was read in Multiskan EX (Thermo Scientific, USA) and measured at 450 nm.

### Statistical analyses

The data are expressed as the mean values ± SD from three independent experiments. Differences between the control and experimental conditions were assessed using different tests. Data with non-normal distribution were analyzed using non-parametrical Mann–Whitney U and Kruskal Wallis tests. When the data attained a normal distribution, Student’s *t*-test, a two-way ANOVA, a multivariate ANOVA and a linear regression test were used. To evaluate the cell viability data, a mixed linear model was used. Statistical significance was considered when the p value was less than 0.05. We performed the analyses using SPSS 15.0 software (IBM SPSS statistics, USA). For the calculations regarding cell viability, free statistical software was used (www.r-project.org) with the nmle package for the mixed linear models.

## Results

### Physical characteristics of the non-cellular post-glycated three-dimensional

**Fig. 1:**
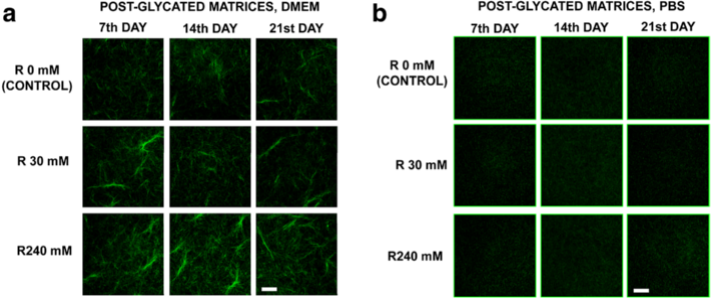
Increased collagen type I fiber reflection fluorescence with non-enzymatic glycation. Collagen type I fiber conformation observed with CRM (laser ex/em 568/568 nm) at different ribose (R) concentration over a period of twenty-one days. **a**. In post-glycated DMEM matrices, collagen type I fibers formed aggregates and reflected more fluorescence with elevated R concentrations. **b**. In post-glycated PBS matrices, collagen type I fibers were short, thin and homogeneous, with no increase of reflected fluorescence with higher R concentration (240 mM). **a** and **b**. Reflection fluorescence of non-glycated matrices (controls) was done to the reflective properties of the collagen. Time seems to not affect the reflection fluorescence of collagen. The increase in CRM signal in DMEM gels could also be contributed by the increased aggregation of collagen fibers observed in these gels, perhaps because the presence of glucose. The scale bar corresponds to 40 μm. CRM = Confocal reflection microscope; DMEM = Dulbecco’s modified Eagle medium; PBS = Phosphate-buffered saline

Using CRM we examined the morphological and structural modifications of type I collagen post-glycated fibers (reflection fluorescence). Different changes occurred depending on the medium used in the preparation of the matrices (DMEM or PBS). DMEM matrices exhibited large, thick fibers that formed aggregates (Fig. 1a), while PBS matrices displayed short, thin collagen fibers with no evidence of aggregation (Fig. 1b). An increased intensity of collagen reflection fluorescence was observed in all the DMEM matrices with the highest ribose concentration (240 mM). However, this phenomenon did not occur in PBS matrices. This different reflection fluorescence intensity in DMEM and PBS matrices was attributed to different pattern of collagen fibers organization depending on the substrate used (DMEM or PBS).

**Fig. 2:**
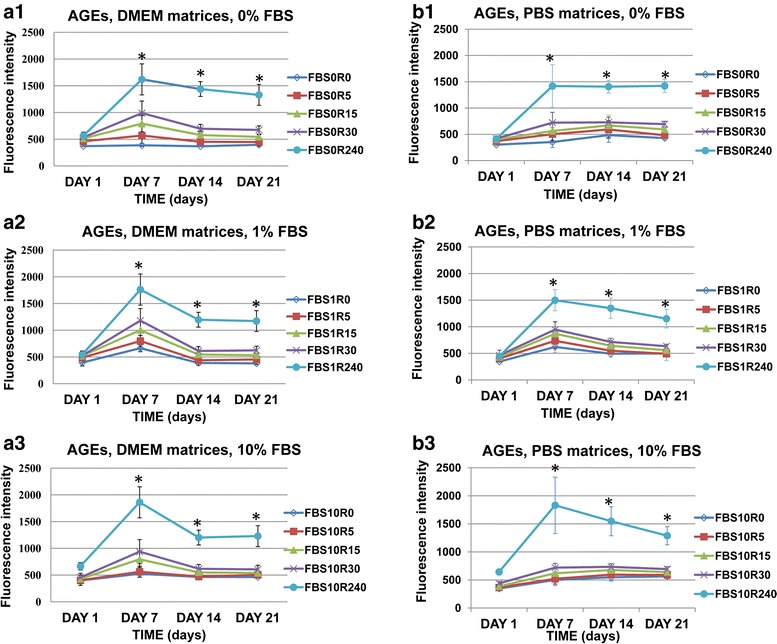
The formation of advanced glycation end products (AGEs) increased with collagen glycation. AGEs formation was evaluated in non-cellular post-glycated DMEM (**a1**-**3**) and PBS (**b1**-**3**) matrices based on autofluorescence (ex/em: 360/440 nm wavelengths). The matrices were elaborated using different concentrations of fetal bovine serum (FBS), 0 % (**a1**, **b1**), 1 % (**a2**, **b2**) and 10 % (**a3**, **b3**). The media was changed every 2 days beginning on the 5^th^ day to allow collagen cross-linking to occur. AGE formation in both type of matrices (DMEM and PBS), was significantly greater with higher ribose concentrations (240 mM) (p < 0.01) and at the 7^th^ day (p < 0.01). After that, less autofluorescence was observed for all the matrices suggesting less AGEs’ formation. Interestingly, DMEM matrices (2A) were more autofluorescent than PBS matrices (2B) at days 1 and 7 (p < 0.05 in practically all conditions). Nevertheless, PBS matrices became more autofluorescent than DMEM ones after the 14^th^ day (p < 0.05 in practically all conditions for FBS 1 % and 10 %). The experiments were repeated three times with similar results obtained. * p < 0.05

**Fig. 3:**
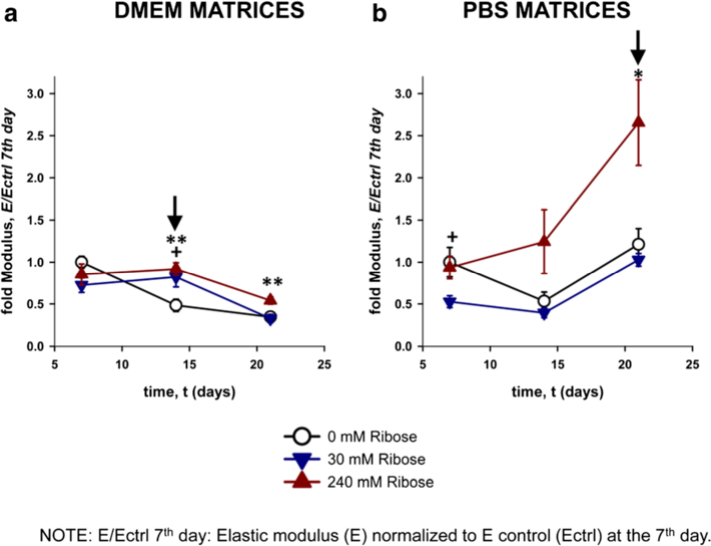
Collagen post-glycation increased the matrix stiffness. The stiffness of the non-cellular post-glycated matrices was measured using atomic force microscopy at days 7, 14 and 21. The values were normalized using that of the non-glycated matrices, i.e. control matrices (0 mM of ribose (R)) at the 7^th^ day. The black arrows indicate the R concentration that rendered the collagen gels stiffer than the non-glycated gel at the 7^th^ day. **a**. DMEM matrices. Collagen gels stiffened in a R dependent manner at the 14^th^ day after glycation at 30 and 240 mM of ribose respect to the control non-glycated gels (p < 0.01). **b**. PBS matrices. The collagen gels stiffened with 240 mM of ribose at the 21^st^ day after glycation (p < 0.01). **a** and **b**. This different stiffening dynamics suggests different cross-linking rates between these gels. The glucose present in DMEM could play a role in the stiffness developed, which could render a higher basal level of glycation before ribose treatment. Accordingly, the non-glycated DMEM gels (controls) showed greater stiffness than the glycated matrices at the 7^th^ day. Phenomenon not observed in PBS matrices. The highest R concentration (240 mM) led to the stiffest conformation of both types of matrices (p < 0.01). p-values for 30 mM of ribose: + (0.01 < p < 0.05), ++ (p < 0.01); p-values for 240 mM of ribose: *(0.01 < p < 0.05), ** (p < 0.01)

To indirectly assess the extent of AGEs formation, we measured the autofluorescence intensity of DMEM and PBS matrices with different FBS concentrations at the wavelength of fluorescent AGEs (ex/em 360/440 nm) by a plate reader as described in the literature [20, 21, 27, 28, 31, 32]. As shown in Fig. 2 (a and b), the content of AGEs increased gradually in a ribose concentration-dependent manner in both conditions, DMEM and PBS (p < 0.05), independently of the serum concentration (p > 0.05). The highest value was found at the highest ribose concentration (240 mM, p < 0.01). An autofluorescence peak was found at 7^th^ day (p < 0.01) in all conditions. Then, less autofluorescence was observed for all the matrices at day 14 and 21, probably due to a decreased amount of AGEs formation after changing the media every 2 days. Interestingly, autofluorescence measurements revealed that DMEM matrices (Fig. 2a) were more autofluorescent than PBS matrices (Fig. 2b) at days 1 and 7 (p < 0.05 in practically all conditions). Nevertheless, PBS matrices became more autofluorescent than DMEM ones after the 14^th^ day (statistically significant in practically all conditions for FBS 1 % and 10 %). Because the accumulation of AGEs generated by non-enzymatic glycation induces modifications and cross-links that stiffen human tissue proteins [22, 25], we examined using the mechanical changes in glycated matrices at the micrometer scale by measuring the Young’s elastic modulus (E) through AFM (Fig. 3). The E value of the control gels (0 mM ribose) at day 7 was 1.6 ± 0.3 and 0.9 ± 0.5 kPa in DMEM and PBS post-glycated matrices (0 % FBS), respectively. All subsequent E values were normalized to the corresponding control values at day 7. Our results revealed that both DMEM and PBS matrices stiffened in a ribose-dependent manner with respect to the control non-glycated gels (p < 0.01). The highest value of stiffness was reached at day 21^st^ for PBS matrices as shown in Table 1 (absolute E value: 2.5 ± 1.35 KPa). Nonetheless, the maximum point of stiffness was not always at the end of the experiment. Fold stiffness in DMEM gels remained rather unaltered after day 14^th^. In contrast we observed a moderate rise in fold gel stiffness in PBS gels up to 21^st^ day (Fig. 3a and b). Consequently, stiffness of the matrix was greater after the 14^th^ day of post-polymerization. However, the basal stiffness values in control gels (0 mM ribose) at day 7^th^ were higher in DMEM than in PBS gels, perhaps because of glucose present in DMEM.

**Table 1:** Summary results from the post-glycated three-dimensional collagen matrices

Types matrices			PBS	DMEM
Fiber collagen I reflection fluorescence (ex/em: 568/568 nm)			Short, thin, homogeny collagen fibers with no evidence of aggregation nor increment of reflection collagen fluorescence.	Large heterogenic thick fibers that formed aggregates. Increase of reflection collagen fluorescence at higher ribose concentration.
Stiffness (absolute values, KPa +/− SD)	7^th^ day	0 mM	0.9 +/− 0.5	1.6 +/−0.3
	14^th^ day	30 mM	0.47 +/− 0.31	1.33 +/− 0.51
		240 mM	1.17 +/−087	1.71 +/−0.72
	21^st^	30 mM	0.97 +/−0.16	0.53 +/−0.21
		240 mM	2.5 +/−1.35	1.03 +/−0.43
Viability	AlamarBlue Assay Fluorescence (ex/em: 550/590 nm)		Data not shown	
			[Ribose]	[Ribose]
			240 mM → cell death	240 mM → cell death
			≤30 mM → ↑ mitochondrial metabolism	≤30 mM → ↑ mitochondrial metabolism
	Confocal Reflection Microscopy, live/dead assay (green/red stain)		Data not shown	
			[Ribose]	[Ribose]
			240 mM → cell death	240 mM → cell death
			≤30 mM → Better viability (green stain)	≤30 mM → Better viability (green stain)
			Cell morphology: round, stellar, short dendritic cells (See Additional file 3).	Cell morphology: spindle, long dendritic cells (See Additional file 3).
Gel contraction			Data not shown	
			[Ribose]	[Ribose]
			Controls (0 mM): from 14^th^ day 5 mM and 15 mM: between 14^th^ and 21^st^ days of culture.	5 mM, 15 mM and controls (0 mM): between 14^th^ and 21^st^ day of culture.
AGEs autoflorescence (ex/em: 360/440 nm)			At ↑[Ribose] → autofluorescence increase.	At ↑[Ribose] → autofluorescence increase.
			Higher autofluorescence at the 14^th^ and 21^st^ day compared with DMEM matrices (*).	Higher autofluorescence at the 1^st^ and 7^th^ day compared with PBS matrices (*).
Protein synthesis	α-smooth muscle actin (α-SMA)		NA	Gene expression (21^st^ day): at 5 (*) and 15 mM of [Ribose] and controls.
				Protein expression (14^th^ and 21^st^ days): in all conditions, particularly with 5 and 15 mM of [Ribose].
	Tenascin-C (TNC)		NA	Protein expression: ↑ in a time-dependent manner at 14 and 21 days (*).

*PBS* phosphate-buffered saline, *DMEM* Dulbecco’s modified Eagle medium, *Ex/em* Excitation/Emission, *nm* nanometers, *KPa* KiloPascals, *SD* Standard deviation, *mM* miliMolar, *N/A* Not Available, [Ribose] concentration of ribose; ↓: decreased; ↑: elevated; ≤: minor or equal; (*) *p* < 0,05

### Effects of post-glycated matrices on primary human normal lung fibroblasts

#### Fibroblast viability

**Fig. 4:**
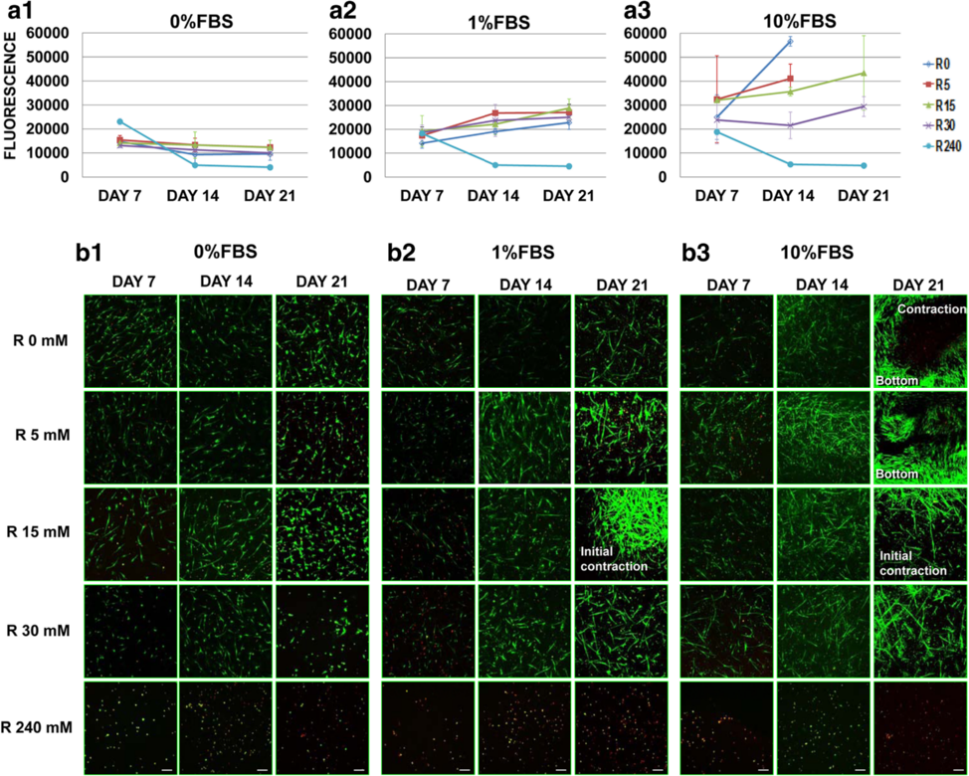
Post-glycated matrices support fibroblast viability for long periods. Primary human lung fibroblasts cultured within different glycated collagen DMEM matrices over a period of 21 days. **a**. Fluorescence determination using alamarBlue assay. **b**. LIVE/DEAD viability/cytotoxicity stained cells observed using confocal reflection microscopy. The viable cells are stained green, and the non-viable cells are stained red. **a** and **b**. Cell death occurred under all conditions at the highest ribose (R) concentration (240 mM), independently of the use of serum (FBS). The use of FBS increased the proliferation rate under the rest of the conditions (p < 0.01). **a1** and **b1** (0 % FBS). No cell proliferation was observed, but cells were alive during the 21 days. **a2** and **b2** (1 % FBS). A gradual increase in cell proliferation was observed under all conditions except R 240 mM, but the rates for R 5 mM (p < 0.05) and the control matrices (p < 0.01) were significantly different only between the 7^th^ and 21^st^ days. **a3** and **b3** (10 % FBS). An improved proliferation rate was observed with the use of 10 % of serum, which was significantly for the control (non-glycated) only between the 7^th^ and 14^th^ days (p < 0.05). Gel contraction was observed in ≤15 mM R conditions between the 14^th^ and the 21^st^ day. No gel contraction was observed at 30 mM R (explanation in discussion). It was not possible to determine the fluorescence in the controls and in matrices glycated using 5 mM R at the 21^st^ day because of gel contraction and cells growing on the bottom of the well (**a3**). The scale bars correspond to 200 μm. The experiments were repeated three times, with similar results obtained

The fibroblast viability in the glycated 3D collagen matrices at different FBS concentrations was determined quantitatively using the AlamarBlue assay, which assesses the extent of mitochondrial activity via fluorescence (redox reaction, Fig. 4a), and confirmed qualitatively by differential staining of live and dead cells visualized using confocal microscopy (Fig. 4b).

In our study, the fluorescence intensity data revealed a marked decrease in fibroblast viability at the highest ribose concentration (240 mM) under all of the gel conditions independently of the substrate FBS content (p < 0.01) (Fig. 4a and b), suggesting a cytotoxic effect of ribose at high concentration. Nevertheless, cells proliferated and survived during 21 days of culture with 5, 15 and 30 mM ribose concentration when 10 % of FBS was used (Fig. 4 a3). No statistical differences in cell viability were observed respect to the control, 0 mM ribose when 0 % of FBS was used (p > 0.05), nor for 1 % too (p > 0.05). A strong effect on cell growth was observed when comparing fibroblasts cultured in matrices with serum with matrices without serum over the period from the 7^th^ to the 21^st^ day, which was independent of the ribose concentration (p < 0.01 for 1 % and 10 % FBS, respectively).

On the other hand, to confirm the results of cell viability and avoid the possible effect of the reducing sugar to the redox reaction of the AlamarBlue assay, data from non-cellular matrices were analyzed (Additional file 4). Results showed that the fluorescence intensity from non-cellular matrices was significantly higher at 240 mM of ribose concentration (p < 0.01) and was independent of the use of serum (p > 0.05) (Additional file 4). Therefore, the observed increase in the fluorescence of the cellular matrices depended on the cell viability, which increased with serum. Then, the best conditions for allowing cell to grow inside the glycated collagen gels up to 21 days are at 5 and 15 mM of ribose and 10 % of FBS.

In addition, images taken using a CRM confirmed that the cells had proliferated, with an increase in the number of viable cells when FBS and low ribose concentrations were used (Fig. 4 b2 and b3) and that cells died with the highest ribose concentration from the first days (data shown in Fig. 4 b1-3 from the 7^th^ day). Moreover, matrix contraction was observed in the lattices with increased cell growth (<15 mM ribose and 10 % FBS) between the 14^th^ and 21^st^ days of culture (Fig. 4 b3).

#### Fibroblast differentiation and synthesis

To evaluate the putative differentiation of fibroblasts to a contractile cell phenotype (myofibroblasts-like), which could contribute to the contraction of the post-glycated DMEM matrices α-smooth muscle actin (α-SMA) gene expression and α-SMA protein levels were examined. Only contracted matrices were evaluated, i.e. those with 10 % FBS. The cells growing on the bottom of the petri dishes were not analyzed (Fig. 4 b3).

**Fig. 5:**
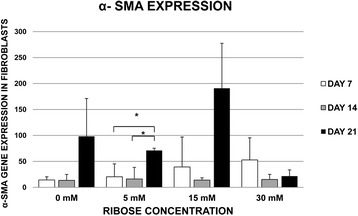
Post-glycated matrices induce a contractile phenotype. Primary human normal lung fibroblasts cultured within three-dimensional glycated DMEM matrices with 10 % of serum were analyzed to determine the level of alpha-smooth muscle actin (α-SMA) gene expression at the 7^th^, 14^th^ and 21^st^ day of culture. α-SMA gene expression was observed in the glycated matrices prepared with 5 mM, 15 mM ribose (R) and in the non-glycated matrices (controls) on the 21st day (only statistically significant (p < 0.05) for 5 mM). This phenomenon corresponded to the occurrence of gel contraction. In contrast, α-SMA gene expression and gels contraction were not observed in the 30 mM R glycated matrices on the 21^st^ day. Interestingly, a progressive increase of α-SMA gene expression was observed with a gradual increase of R on day 7^th^, suggesting that the differentiation of fibroblasts to a contractile phenotype depends not only on time but also on the ribose concentration, which is associated with the extent of collagen cross-linking. The data are presented as percentages respect to the values on the 1^st^ day. The experiments were repeated three times, with similar results obtained. * p-value < 0.05

**Fig. 6:**
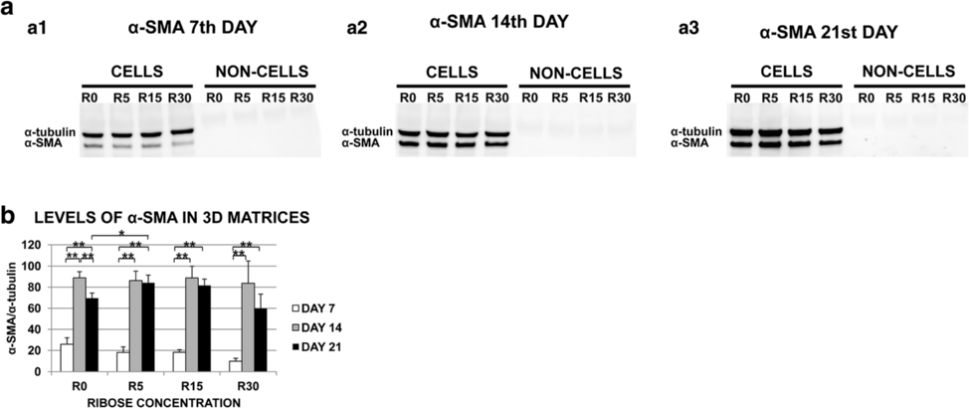
α-SMA protein detection in the 3D matrices. alpha-smooth muscle actin (α-SMA) protein expression in the primary human normal lung fibroblasts was evaluated using western blot. Fibroblasts were cultured within 3D glycated DMEM matrices containing 10 % serum. **a**. Western Blot. α-SMA band, 42 kDa; α-tubulin band, 50 kDa. Albumin band (66 kDa, due to FBS and visible in cellular and acellular matrices blots’, probably because of the difficulty in rinsing all the media from the matrices prior to protein extraction). The experiments were repeated three times. **a1.** An insignificant amount of α-SMA was detected in all the cellular matrices. **a2** and **a3.** Higher amounts of α-SMA were observed in all the cellular matrices on the 14^th^ and 21^st^ days of culture. **a1, a2** and **a3.** No tubulin or α-SMA bands were detected in the non-cells matrices. **b**. Densitometric analysis of α-SMA levels (Ratio of α-SMA to α-tubulin). The results were obtained from three independent experiments. *p < 0.05, **p < 0.01. A gradual decrease of α-SMA was observed at higher ribose concentrations on the 7^th^ day of culture (p > 0.05). Contractile phenotype was most strongly detected on the 14^th^ and 21^st^ days of culture. It was significantly higher at all ribose concentrations, also for controls without ribose, compared with the level on the 7^th^ day. It suggests that the microambient ‘per se’ could induce a phenotype change and that cells contribute to the matrix contraction

A higher increase of α-SMA gene expression was observed in 0–15 mM of ribose at the 21^st^ day (Fig. 5). α-SMA protein detection appeared from day 7^th^ in all conditions, with and without ribose, and was significantly increased at days 14 and 21 compared to the 7^th^ day (p < 0.01), which suggests that the induction of α-SMA is modified by the 3D microambient ‘per se’ (Fig. 6).

**Fig. 7:**
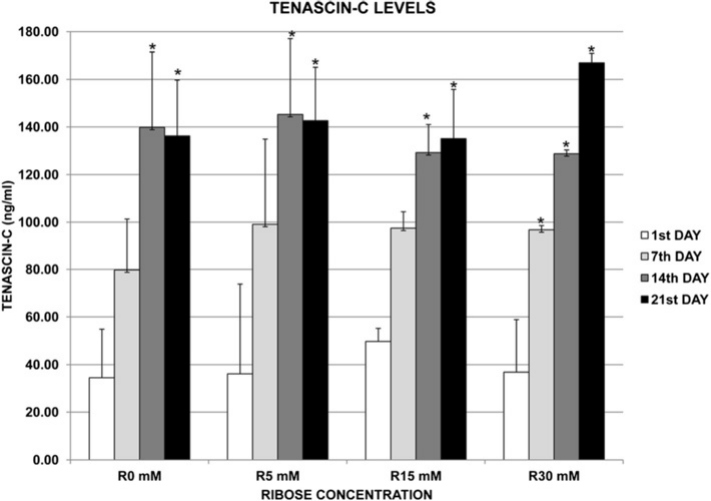
3D matrices induce Tenascin-C synthesis in lung fibroblasts. Levels of Tenascin-C (TNC) were measured from the supernatants of the 3D DMEM matrices by ELISA. The results were obtained from three independent experiments. Fibroblasts were cultured with 10 % of FBS. The synthesis of TNC was induced in a time-dependent manner, observing significant statistical differences between the 1^st^ and the 14^th^ and 21^st^ days of culture (p < 0.05 for both comparisons). Although no differences were appreciated between the 14^th^ and the 21^st^ days of culture, a higher level of TNC was observed with 30 mM of ribose at the 21^st^ day (p = 0.08). No statistical differences were observed between glycated and non-glycated matrices. These results suggest that the 3D microenvironment could be enough to produce changes in the secretion of TNC by normal fibroblasts in a time-dependent manner

The supernatants of the DMEM matrices from three independent experiments were analyzed to evaluate the fibroblast tenascin-C (TNC) protein synthesis (Fig. 7). Results showed a progressive increase in the secretion of TNC in all the matrices from the 1^st^ to the 21^st^ day, with statistical differences between the 1^st^ and the 14^th^ day and the 1^st^ and the 21^st^ day (p < 0.05 for both comparisons). Although no differences were appreciated between the 14^th^ and the 21^st^ day of culture in most ribose conditions, a higher level of TNC at the 21^st^ day was observed with 30 mM of ribose (p = 0.08). No statistical differences were found between ribosilated and non-ribosilated matrices. These results suggest that the 3D microenvironment could be enough to produce changes in the secretion of TNC by normal fibroblasts in a time-dependent manner.

## Discussion

Lung tissue fibroblasts are normally surrounded by a 3D ECM and the interaction between fibroblasts and ECM proteins could be crucial in tissue homeostasis and disease. However, most *in vitro* studies in lung fibrosis have been performed with fibroblasts growing in a plate (2D) or in 3D based on polyacrylamide hydrogels [8, 38, 39] or collagen type I gels [17, 18, 27, 40] with fibroblasts growing on top. Few studies have analyzed the viability of fibroblasts growing within type I collagen matrices [7, 41]. Our study was designed to modify the 3D collagen matrix via non-enzymatic glycation and to analyze the impact of such matrices on fibroblast phenotype and viability growing inside the gel. To our knowledge, there are no previous reports of analyzing fibroblasts viability in glycated DMEM matrices by using two corroborating cell growth methods. We used ribose as a reducing sugar rather than glucose due to the ability of ribose to modify faster the physical and chemical properties of type I collagen gels [21]. Very few studies have used post-glycated collagen matrices with cells growing inside, and none in the field of lung fibrosis [19, 21]. Our novel 3D culture model displayed collagen enrichment and an aging/oxidative microenvironment with increased stiffness, which may be useful for the study of cell behavior and phenotypic changes that depend on pro-fibrotic ECM conditions. Our study points up the best three-dimensional ribosilated matrices conditions to allow primary lung fibroblast viability and growth. Furthermore, the results demonstrate that the 3D collagenated matrices induce fibroblast-to-contractile phenotype differentiation and an increase of TNC synthesis. We observed structural differences in the collagen fibers depending on the medium used in the preparation of the matrices (DMEM or PBS), which may be attributable to the presence of glucose in DMEM. Our results showed that once the matrices have been glycated, the higher ribose concentrations (240 mM) increased the reflectance fluorescence of collagen type I in DMEM matrices (Fig. 1) and the AGEs production (Fig. 2) in agreement with previous published results [20, 21, 27]. However, the ribose-dependent increase in CRM signal in DMEM gels could also be contributed by the increased aggregation of collagen fibers observed in these gels, which could locally enhance the reflective properties of the collagen. In contrast, such structure-related differences observed by CRM may not contribute to the autofluorescence measured by the plate reader or to the stiffness results.

The interest of using glycated collagen gels was to resemble the increased stiffness observed in aged and fibrotic lungs [2, 42]. Furthermore, the use of the post-glycation method (instead of pre-glycation technique) aimed to approach to the collagen cross-links, which could occur *in vivo*. Some studies have used non-enzymatic glycation to create stiffer scaffolds with increased mechanical rigidity but none concerned to lung fibrosis [20, 21, 27, 43]. Our results demonstrated that the glycation process stiffened the collagen matrices in a concentration-dependent manner, occurring earlier in the DMEM matrices than in the PBS matrices. To our knowledge, we provide first evidence that glycation-induced collagen gel stiffening depends not only on ribose concentration, but also on the type of substrate solution used (DMEM or PBS). However, these different stiffening dynamics, suggests different cross-linking rates between those gels (DMEM compared to PBS), perhaps attributed to the glucose already present in DMEM, which could render a higher basal level of glycation before ribose treatment and to the diameter and length of the cross-linked collagen fibrils [42]. In support of this hypothesis, the basal stiffness values in control gels (0 mM of ribose) at day 7^th^ were higher in DMEM than in PBS gels. Our stiffness values were similar to those reported by Roy et al. [20], and slightly higher than those from other authors without cells [27, 43], most likely because of differences in the experimental conditions, such as the method of measure. In contrast, when cells were cultured within the glycated matrices, an elevated Young modulus was observed [19, 21]. Interestingly, a recent study by Booth et al. [14] showed an increased stiffness of decellularized fibrotic lungs compared with normal lungs, plausibly attributed to the substantial and varied amounts of pro-fibrotic ECM proteins present in the former in addition to collagen. Thus, it is conceivable that cells embedded into the collagen gels would also upregulate the expression of other interstitial proteins, increasing the rigidity of the gel, which will be the focus of future *in vitro* studies [3, 42]. As a preliminary data, the fibroblasts growing inside our 3D collagen matrices increased the synthesis of TNC, a pro-fibrotic ECM protein. This phenomenon occurred in a time-dependent manner, independently of the ribose concentration. However, the level of TNC was higher in 30 mM ribosilated matrices at the 21^st^ day, suggesting that TNC synthesis by normal fibroblasts was dependent on the cell-type I collagen interactions and also on the indirect effect of ribose on collagen (stiffness, AGEs or collagen cross-links). These observations leads to further studies in order to better explain this effect.

Importantly, the non-enzymatic glycation of collagen resulted in the formation of AGEs, which are related to aging and have been observed to stiffen tissues in different degenerative diseases [22–25]. With age, collagen becomes less soluble [26], more cross-linked and more glycosylated [5]. In studies of aged collagen fibers from different tissues, such as skin and lungs, the presence of AGEs was detected indirectly as an increase in the collagen autofluorescence [31, 32]. Our results showed that the change in the autofluorescence of glycated collagen, measured with the specific wavelength of fluorescent AGEs as reported elsewhere [20, 21, 27, 28, 31, 32], was dependent on the formation of AGEs (Fig. 2). Furthermore, we have shown that the collagen autofluorescence was affected by the ribose concentration in a dose-dependent manner, with fluorescence increasing when the highest ribose concentration was used. In support of our findings, other groups have reported that the fluorescence of glycated collagen matrices gradually increased with increasing concentrations of reducing sugars independently of the carbohydrate used for glycation [20, 21, 27].

On the other hand, a peak of autofluorescence was always observed at 7th day and then the autofluorescence decreased (days 14^th^ and 21^st^), probably because less new AGEs formation after day 7^th^. It could be a consequence of the technical approach, since the media was not changed until the 5th day after post-glycation and then it was changed every two days. This phenomenon could be attributed to the rinse of the matrices with the changes of the media, which would attenuate the formation of new cross-links between the glucose and the collagen. Another possibility would be that the cross-links could be higher at the beginning of the post-glycated matrices reaction. Additionally, our experiments of autofluorescence in PBS and DMEM matrices revealed different cross-linking rates. As DMEM matrices (Fig. 2a) showed more autofluorescence than PBS matrices (Fig. 2b) at days 1 and 7, less autofluorescence from DMEM compared to PBS matrices was observed since the 14^th^ day. These different cross-linking rates could explain the differences in stiffening dynamics between those gels (DMEM and PBS). In agreement with these dynamics, fold stiffness in DMEM gels remained rather unaltered after day 14th. In contrast we observed a moderate rise in fold gel stiffness in PBS gels to the 21st day.

One of the main findings in the present study was the demonstration of the profound effect of the physical and chemical changes of the three-dimensional matrices on the fibroblast phenotype and cell growth. We demonstrated that fibroblasts embedded in the matrices containing low ribose concentrations (≤30 mM) were able to proliferate and that the better growth rate was observed using FBS, whereas significant cell mortality was observed in matrices with highest ribose concentration (240 mM) independently of the use of FBS (Fig. 4), most likely because of the increased osmosis [19]. Remarkably, we detected a contractile cell phenotype expressing alpha-smooth muscle actin (α-SMA). This occurrence of the contractile phenotype was related to the contraction of glycated gels using 5 and 15 mM of ribose and to that of the non-glycated matrices between the 14^th^ and 21^st^ days, when 10 % of serum was used (Fig. 4 b3). Remarkably α-SMA was induced without adding TGFβ1, suggesting that the microenvironment ‘per se’ modifies cell phenotype by increasing cell contractility. Therefore, the experiments of expression and detection of α-SMA as a contractile phenotype marker (Figs. 5 and 6, respectively), were performed only in the matrices where the gel contraction was observed, (i.e. when 10 % of FBS was used), to verify if the cells embedded into the matrices could contribute to their contraction. Therefore, it is important to emphasize two messages: 1) the induction of α-SMA expression and protein level is modified by the microambient and 2) cells contribute to the matrix contraction.

The mechanisms by which cells contract the collagen gels are unclear. Based on previous studies, it is conceivable that as matrix stiffness increases, the fibroblasts develop proportional isometric tension, acquiring prominent actin stress fibers that cause the contraction of the matrices [3, 5, 7, 17, 44, 45]. Nevertheless, fibroblasts that do not surmount the tension of the matrix also develop actin stress fibers, but they do not contract the lattice [7, 17]. This attribute is relevant to the differences found in α-SMA detection and the contraction of our gels depending on the ribose concentration, as occurs with 30 mM of ribose [7, 44]. In those matrices, the contractile phenotype, α-SMA positive (Fig. 6 a2 and a3), did not contract the gels (Fig. 4 b3), probably because the cells did not surmount the tension present in the matrix.

Thus, matrix contraction depends on the presence of fibroblasts [7, 15, 45, 46], the use of serum [7, 44, 46], and the glycation cross-linking [17]. Additionally, the degree of contraction also depends on the initial collagen concentration [15], the number of cell passages [7, 15, 46] and the temperature [46].

Even though, we have not performed stiffness experiments when cells are seeded into the matrices, our results suggests that the effect of the matrix on the cells could be because of the matrix biomechanics’ microenvironment, the ECM protein cross-links or even either microenvironment factors that can be produced under these conditions. In support of this interpretation, other authors have reported that an increase in the ECM protein concentration, as well as the matrix cross-linking and the mechanical interactions between the ECM and the cells, promote cell proliferation, the acquisition of a contractile phenotype and the modulation of gel contractility, which they attribute to the matrix stiffness [4–8, 15, 17, 38, 40, 42, 43].

## Conclusions

Human lung fibroblasts are able to grow into 3D collagenated and stiffened matrices under specific conditions. The increase of ECM cross-linking via non-enzymatic glycation at low ribose concentrations and the biomechanical interactions between the ECM and the cells embedded in it induces fibroblast phenotype and metabolic changes. Altogether could modulate the gels’ contractility. This applicable model with a mechanic and oxidative modified environment could resemble an aging/fibrotic ECM. Accordingly, such model may be useful for investigating cell behavior and phenotypic changes that occur during the processes of lung fibrosis and aging, as well as for testing different antifibrotic therapies *in vitro*.

Table 1. Summary results from the post-glycated three-dimensional collagen matrices. PBS: phosphate-buffered saline; DMEM: Dulbecco’s modified Eagle medium; ex/em: excitation/emission; nm: nanometers; KPa: KiloPascals; SD: Standard deviation; mM: miliMolar; N/A: Not Available; [Ribose]: concentration of ribose; ↓: decreased; ↑: elevated; ≤: minor or equal; (*) p < 0,05.

## Additional files

Additional file 1:
**Markers of primary human normal lung fibroblast.** The primary human normal lung fibroblasts used in this study at passage 3 were analyzed by western blot. a. Tenascin-C (+), E-Cadherin (−), Vimentin (+++) and β-actin (+++). b. α-tubulin (++) and α-SMA (+). Additional file 2:
**Movie of fibroblasts distribution when cultured into a 3D collagen matrix.** Green cells are live cells. Red cells are dead cells. Additional file 3:
**Fibroblasts morphology in collagen 3D matrices.** Different fibroblast morphology was appreciated into 3D collagen matrices depending on the media conditions (DMEM vs PBS). While with DMEM the fibroblasts were as long dendritic cells and spindle; in PBS, the cells appear round, stellar, and as short dendritic cells. Additional file 4:
**Fluorescence in cell-free glycated matrices.** Fluorescence intensity increased with higher ribose concentrations under all conditions (a, b and c) and was independent of the presence of serum (b and c).

## Abbreviations

3D: Three-dimensional
AFM: Atomic force microscopy
AGEs: Advanced glycation end products
BSA: Bovine serum albumin
CRM: Confocal Reflection Microscopy
DMEM: Dulbecco’s modified Eagle medium
DNA: Deoxyribonucleic acid
E: Young’s modulus
ECM: Extracellular matrix
FBS: Fetal bovine serum
IPF: Idiopathic Pulmonary Fibrosis
mM: mili-Molar
PBS: Phosphate-buffered saline
qPCR: Real-time quantitative reverse transcription-polymerase chain reaction
RIPA: Radioimmunoprecipitation assay buffer
RNA: Ribonucleic acid
TNC: Tenascin-C
α-SMA: Alpha-smooth muscle actin
