# Erratum to: Fibroblast viability and phenotypic changes within glycated stiffened three-dimensional collagen matrices

**DOI:** 10.1186/s12931-015-0277-4

**Published:** 2015-11-02

**Authors:** Vanesa Vicens-Zygmunt, Susanna Estany, Adai Colom, Ana Montes-Worboys, Carlos Machahua, Andrea Juliana Sanabria, Roger Llatjos, Ignacio Escobar, Frederic Manresa, Jordi Dorca, Daniel Navajas, Jordi Alcaraz, Maria Molina-Molina

**Affiliations:** Department of Pneumology, Unit of Interstitial Lung Diseases, University Hospital of Bellvitge, Barcelona, Spain; Pneumology Research Group, IDIBELL, University of Barcelona, Barcelona, Spain; Unit of Biophysics and Bioengineering, University of Barcelona, Barcelona, Spain; Department of Preventive Medicine, University Hospital of Bellvitge, Barcelona, Spain; Department of Pathology, University Hospital of Bellvitge, Barcelona, Spain; Department of Thoracic Surgery, University Hospital of Bellvitge, Barcelona, Spain; Research Network in Respiratory Diseases (Centro de Investigación Biomédica en Red (CIBER) de Enfermedades Respiratorias), ISCIII, Barcelona, Spain; Department of Biochemistry, University of Geneva, Science II, Geneva, Switzerland

## Erratum

After publication of the original article [1] it came to the publisher’s attention that there was a formatting error in Table 1 and the text ‘5 mM and 15 mM’ had been inserted on the wrong line. This has now been corrected in the original article.
