# Supplementary figures and images for: Fibroblast viability and phenotypic changes within glycated stiffened three-dimensional collagen matrices
Source: Respir Res. 2015 Jul 1;16(1):82. doi: 10.1186/s12931-015-0237-z (PMC4494165; doi:10.1186/s12931-015-0237-z)

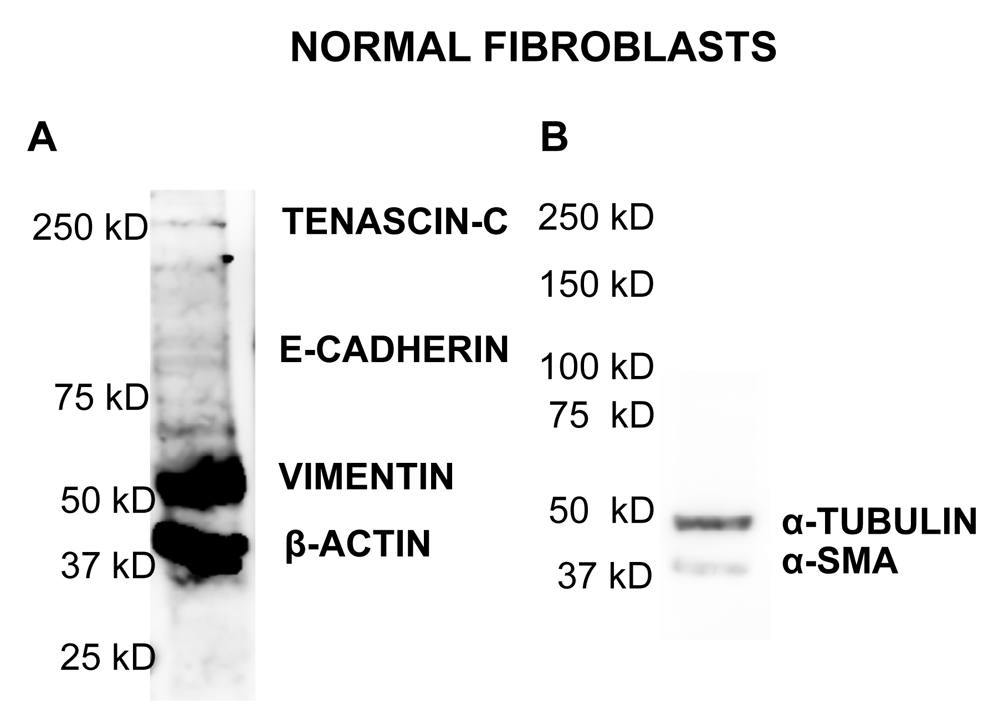

Supplement: Additional file 1: — Markers of primary human normal lung fibroblast. The primary human normal lung fibroblasts used in this study at passage 3 were analyzed by western blot. a. Tenascin-C (+), E-Cadherin (−), Vimentin (+++) and β-actin (+++). b. α-tubulin (++) and α-SMA (+). [file 12931_2015_237_MOESM1_ESM.tif]

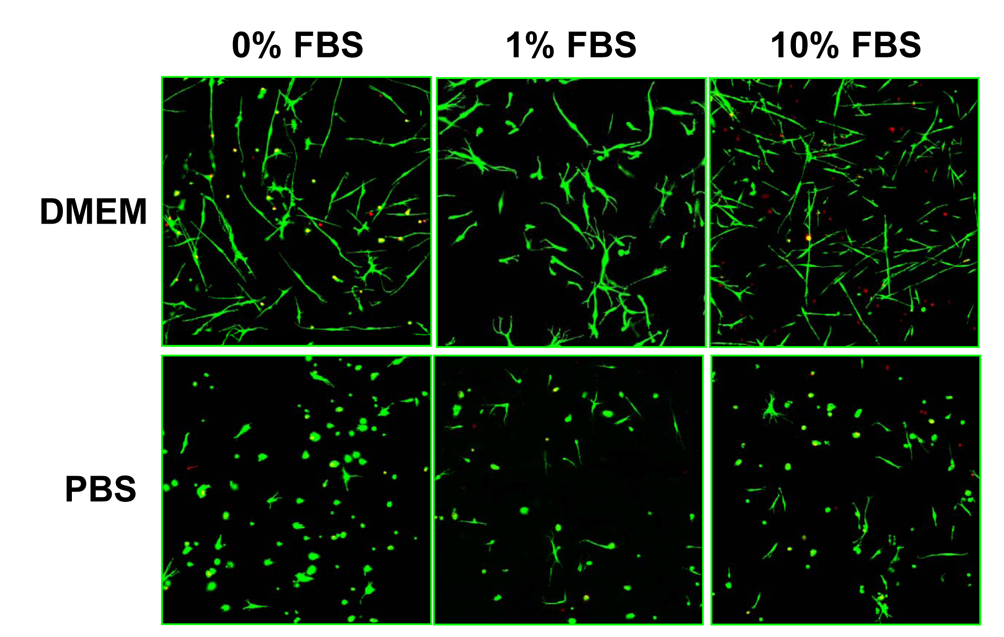

Supplement: Additional file 3: — Fibroblasts morphology in collagen 3D matrices. Different fibroblast morphology was appreciated into 3D collagen matrices depending on the media conditions (DMEM vs PBS). While with DMEM the fibroblasts were as long dendritic cells and spindle; in PBS, the cells appear round, stellar, and as short dendritic cells. [file 12931_2015_237_MOESM3_ESM.tif]

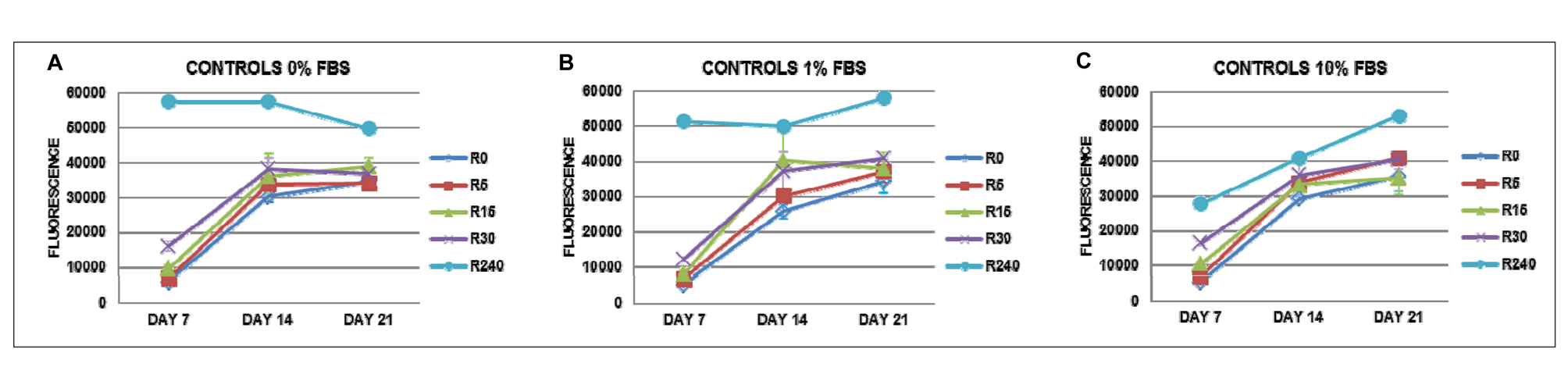

Supplement: Additional file 4: — Fluorescence in cell-free glycated matrices. Fluorescence intensity increased with higher ribose concentrations under all conditions (a, b and c) and was independent of the presence of serum (b and c). [file 12931_2015_237_MOESM4_ESM.tif]
